# Arterial stiffness, endothelial and cognitive function in subjects with type 2 diabetes in accordance with absence or presence of diabetic foot syndrome

**DOI:** 10.1186/s12933-016-0483-5

**Published:** 2017-01-06

**Authors:** Antonino Tuttolomondo, Alessandra Casuccio, Giovanni Guercio, Carlo Maida, Alessandro Del Cuore, Domenico Di Raimondo, Irene Simonetta, Danilo Di Bona, Rosaria Pecoraro, Vittoriano Della Corte, Eliana Gulotta, Gaspare Gulotta, Antonio Pinto

**Affiliations:** 1 Dipartimento Biomedico di Medicina Interna e Specialistica (Di.Bi.M.I.S), University of Palermo, Palermo, Italy; 2 Department of Sciences for Health Promotion and Mother Child, University of Palermo, Palermo, Italy; 3 Dipartimento di Chirurgia Generale e d’Urgenza, Policlinico Universitario “Paolo GiacconeUniversity of Palermo, Palermo, Italy; 4 Chair of Allergology, University of Bari, Bari, Italy; 5 U.O.C di Medicina Interna con Stroke Care, Dipartimento Biomedico di Medicina Interna e Specialistica (Di.Bi.M.I.S), University of Palermo, P.zza delle Cliniche, n.2, 90127 Palermo, Italy

**Keywords:** Endothelium function, Vascular stiffness, Foot ulcer, Vascular health, Cognitive function

## Abstract

**Background:**

Endothelial dysfunction is an early marker of cardiovascular disease so endothelial and arterial stiffness indexes are good indicators of vascular health. We aimed to assess whether the presence of diabetic foot is associated with arterial stiffness and endothelial function impairment.

**Methods:**

We studied 50 subjects with type 2 diabetes mellitus and diabetic foot syndrome (DFS) compared to 50 diabetic subjects without diabetic foot, and 53 patients without diabetes mellitus, by means of the mini mental state examination (MMSE) administered to evaluate cognitive performance. Carotid-femoral pulse wave velocity (PWV) and augmentation index (Aix) were also evaluated by Applanation tonometry (SphygmoCor version 7.1), and the RH-PAT data were digitally analyzed online by Endo-PAT2000 using reactive hyperemia index (RHI) values.

**Results:**

In comparison to diabetic subjects without diabetic foot the subjects with diabetic foot had higher mean values of PWV, lower mean values of RHI, and lower mean MMSE. At multinomial logistic regression PWV and RHI were significantly associated with diabetic foot presence, whereas ROC curve analysis had good sensitivity and specificity in arterial PWV and RHI for diabetic foot presence.

**Conclusions:**

Pulse wave velocity and augmentation index, mean RHI values, and mean MMSE were effective indicators of diabetic foot. Future research could address these issues by means of longitudinal studies to evaluate cardiovascular event incidence in relation to arterial stiffness, endothelial and cognitive markers.

## Background

Diabetic foot syndrome (DFS) or disease (DFD) includes several pathologies, mainly diabetic peripheral neuropathy and peripheral arterial disease which result in foot ulceration [1, 2].

Recent studies [3–5] have highlighted the association between the occurrence of diabetic foot ulcer and microangiopathy complications, including albuminuria (Alb) and diabetic retinopathy (DR), thus underlying the pathogenic role of vascular disease in DFS pathogenesis [5–11]. Understanding of the pathophysiology of atherosclerosis and its vascular complications has increased dramatically, developing numerous measurable biomarkers that play a role in the atherosclerosis process, and are associated with clinically important vascular events. Putative measurable variables include soluble biomarkers and imaging assessments of vascular structure and function.

Our group reported [12] that compared to diabetics without diabetic foot, diabetic subjects with diabetic foot had higher IL-6 and resistin plasma levels, and lower adiponectin plasma levels, as possible determinants of higher cardiovascular risk. Endothelial dysfunction is an early marker of vascular diseases, and methods of assessing endothelial function and arterial stiffness are available for clinical research confirming that endothelial function and arterial stiffness markers are surrogate cardiovascular markers [13].

Endothelial dysfunction in peripheral arteries is assessed by forearm flow-mediated vasodilation [13]. However, the results of forearm flow-mediated vasodilation can vary due to technical problems encountered during measurement, and thus forearm flow-mediated vasodilation is not standardized among institutions [14]. Kuvin et al. [15] described a new method to evaluate endothelial dysfunction called reactive hyperemia peripheral arterial tonometry (RH-PAT). It is a noninvasive, automatic, and quantitative clinical test for digital measurement of hyperemic response, and inversely correlates with various cardiovascular risk factors [16] indicating the practical usefulness of the RH-PAT test.

Considering the reported link between diabetic foot and cardiovascular risk and consistent with the role of putative cardiovascular surrogate markers of arterial stiffness and endothelial function indexes, we aimed to assess whether the presence of diabetic foot is associated with arterial stiffness and endothelial function index impairment in a group of patients with diabetic foot syndrome compared to age matched diabetic subjects without foot complications and age-matched healthy subjects.

## Methods

We recruited all subjects with type 2 diabetes mellitus and foot ulceration referred to the Diabetic Foot Intervention Clinical Group of Policlinico “P. Giaccone” Hospital of the University of Palermo, Italy from September 2014 to December 2015; we also recruited diabetic subjects without diabetic foot admitted from September 2014 to December 2015 for every condition related to diabetic disease (decompensated diabetes, hypoglycemia, but not for new vascular events) in the U.O.C di Medicina Interna con Stroke Care of University Policlinico “P. Giaccone” Hospital of Palermo, and consecutive patients without diabetes mellitus admitted to our wards for causes other than diabetic foot and acute cardiovascular events between 2013 and 2015. The study was carried out in accordance with the principles of the Declaration of Helsinki as revised in 2001. All patients gave informed consent to take part in this research. We obtained consent for publication from the participant (or legal parent) to report individual patient data.

Diabetic foot syndrome is defined, according to the World Health Organization, as “ulceration of the foot (distally from the ankle and including the ankle) associated with neuropathy and different grades of ischemia and infection [17]. Foot ulcer was defined as a full-thickness skin defect that required ≥14 days for healing [18]. Every subject with diabetic foot was matched for age (±3 years) and sex, with one subject without diabetic foot and one healthy subject.

Patients with inflammatory or infectious diseases, autoimmune and rheumatic diseases, cancer, hematological diseases and severe renal or liver failure, as well as those who were under treatment with anti-inflammatory drugs, were excluded. We also excluded patients with fever and recent venous thromboembolism. A physical examination with emphasis on the lower limbs was performed by researchers, who assessed the presence of the following characteristics: hammer/claw toe, Charcot deformity, hallux limitus, prominent metatarsal heads, hallux valgus, bony prominences, and ankle and halluxobility measured by goniometry.

Diabetic peripheral neuropathy was evaluated by careful patient history review and physical examination of the feet using the combination of patient’s neuropathic symptoms, clinical signs and diagnostic tests. To assess the neuropathic symptoms we used the Neuropathy Symptom Score (NSS) [19], which is widely used in clinical practice and has shown high validity and sensitivity. Semmes–Weinstein monofilament was the tool used for the assessment of the diabetic peripheral neuropath [20]. Diagnosis of type 2 diabetes was based on the revised criteria of the American Diabetes Association, using a value of fasting blood glucose ≥126 mg/dl, or it was determined using a clinically based algorithm that considered age at onset, presenting weight and symptoms, family history, onset of insulin treatment, and history of ketoacidosis [21]. Hypertension was defined according to the 2013 ESC–ESH criteria [22]. Dyslipidemia was defined as TG level ≥150 mg/dl and LDL cholesterol >100 mg/dl and HDL cholesterol level <40 mg/dl (regardless of patient’s gender) [23].

### Clinical and laboratory assessment

Clinical and anthropometric data were collected at the time of enrollment. Subjects were classified as normal weight (BMI 18.5–24.9 kg/m^2^), overweight (BMI 25–29.9), or obese (BMI ≥30). A 12-h overnight fasting blood sample was drawn at the time of enrollment to determine the serum levels of ALT, total cholesterol, HDL-cholesterol, triglycerides, and plasma glucose.

### Assessment of cognitive function (mini mental state examination)

The mini mental state examination (MMSE)—a tool that can be used to systematically and thoroughly assess mental status—was administered. It is an 11-question measure that tests 5 areas of cognitive function (orientation, registration, attention and calculation, recall, and language). The maximum score is 30 whereas a MMSE value <24 (23 or lower) is indicative of cognitive impairment [24].

### PWV measurement

Carotid-femoral PWV was measured in the supine position using the automatic device (SphygmoCor version 7.1) that measured the time delay between the rapid upstroke of the carotid and femoral artery pulse waves. The distance between the 2 arterial points was measured on the surface of the body using a tape measure. PWV was calculated as the distance traveled by the arterial pulse wave (in meters) divided by the time delay between the 2 arterial points (in seconds), thus expressed as meters per second.

### Pulse wave analysis

Applanation tonometry was used to record radial artery pressure waveform continuously, and mean values of ≥2 screens of pulse waves of good quality were used for analysis. On the basis of the collected data, an averaged radial pressure waveform was generated and a corresponding aortic pressure waveform and BP were calculated by the validated transfer function (SphygmoCor version 7.1). The aortic pressure waveform was used to calculate the AIx (difference in height between the first and second systolic peaks expressed as a percentage of PP).

### Rh-pat

The principle of RH-PAT has been described previously by researchers [16]. Briefly, a blood pressure cuff was placed on 1 upper arm, while the contralateral arm served as a control. PAT probes were placed on 1 finger of each hand. After a 5-min equilibration period, the cuff was inflated to 60 mm Hg above the systolic pressure or 200 mm Hg for 5 min and then deflated to induce reactive hyperemia. The RH-PAT data were digitally analyzed online (Endo-PAT2000 software version 3.0.4). The RH-PAT index reflects the extent of reactive hyperemia and was calculated as the ratio of the average amplitude of PAT signal over 1 min starting 1.5 min after cuff deflation (control arm, A; occluded arm, C) divided by the average amplitude of the PAT signal of a 2.5-min time period before cuff inflation (baseline) (control arm, B; occluded arm, D). Thus RH-PAT index (RHI) = (C/D)/(A/B) × baseline correction.

## Statistical analysis

Statistical analysis of quantitative and qualitative data, including descriptive statistics, was performed for all items. Continuous data are expressed as mean ± SD, unless otherwise specified. Baseline differences between groups were assessed by the Chi square test or Fisher exact test, as needed for categorical variables, and by the univariate analysis of variance (ANOVA) for parametric variables. Multinomial logistic regression analysis examined the correlation between patient characteristics (independent variables), and patient groups (dependent variable) in a multiple regression model. Odds ratios (OR) and their 95% confidence intervals (CIs) were also calculated and adjusted for drug therapy as covariate. We also adjusted for other clinical variables such as BMI and BSA. To assess the predictive rate of different cutoff values of PWV, Aix and RHI values with regard to patient groups, a receiver operating characteristic (ROC) curve with calculations of area under the curve and 95% CI was constructed, and sensitivity and specificity values were calculated. Spearman correlation analysis was conducted to examine the association between RHI, arterial stiffness indexes, MMSE and other clinical and laboratory variables in patient groups. Data were analyzed by the Epi Info software (version 6.0, Centers for Disease Control and Prevention, Atlanta, GA, USA), and SPSS Software 22.0 version (IBM Corp., Armonk, NY, USA). All P-values were two-sided, and P-values less than 0.05 were considered statistically significant.

## Results

We enrolled 50 subjects with diabetic foot, 50 diabetic subjects without diabetic foot and 53 healthy subjects. General and laboratory variables of diabetic patients with diabetic foot and healthy subjects are listed in Table 1. In comparison to diabetic subjects without diabetic foot, patients with diabetic foot were more likely to have higher systolic blood pressure (135.0 ± 21.8 mm/Hg vs. 124.5 ± 16.8 vs. 116.3 ± 13.4, P < 0.0001), higher BMI (30.2 ± 6.4 vs. 29.9 ± 4.5 vs. 25.1 ± 4.3; P < 0.0001), higher frequency of hypertension (92 vs. 62 vs. 41%; P < 0.0001), higher frequency of previous cardiovascular events (24 vs. 1 vs. 0%; P < 0.0001), and higher frequency of dyslipidaemia (70 vs. 44 vs. 15%; P < 0.0001) (see Table 1).

**Table 1:** Demographic, clinical and laboratory variables in subjects with diabetic foot and in controls (diabetic subjects without diabetic foot and subjects without diabetes)

	Subjects with diabetic foot (n: 50)	Diabetic subjects without diabetic foot (n: 50)	Healthy controls (n: 53)	p
M/F (n/%)	35/15 (70/30)	27/23 (54/46)	26/27 (49.1/50.9)	0.087
Age (years) (mean ± SD)	61.6 ± 10.1	60.6 ± 12.5	63.0 ± 13.9	0.71
SBP (mm/hg) (mean ± SD)	135.0 ± 21.8	124.5 ± 16.8	116.3 ± 13.4	<*0.0001*
DBP (mm/hg) (mean ± SD)	67.9 ± 10.7	70.9 ± 11.2	71.3 ± 12.7	0.283
BMI (kg/m^2^) (mean ± SD)	30.2 ± 6.4	29.9 ± 4.5	25.1 ± 4.3	<*0.0001*
BSA (mean ± SD)	2.02 ± 0.23	1.97 ± 0.25	1.79 ± 0.23	<*0.0001*
Creatinin (mg/dl) (mean ± SD)	1.05–0.27	1.01 ± 0.27	0.90 ± 0.11	*0.008*
EGFR (ml/min) (mean ± SD)	73.46 ± 13.48	73.38 ± 13.53	92.59 ± 6.06	<*0.0001*
Micro albuminuria (n/ %)	12	14	0	<0.0001
Hypertension (n/%)	46 (92)	31 (62)	5 (9)	<*0.0001*
Previous cardiovascular events (n/%)	12 (24)	5 (1)	0 (0)	<*0.0001*
Previous stroke (n/%)	3 (6)	2 (4)	0 (0)	0.202
Dyslipidaemia (n/%)	35 (70)	22 (44)	8 (15.1)	<*0.0001*
AIX (%) (mean ± SD)	139.1 ± 19.2/27.3 ± 9.8	145.0 ± 27.5/29.0 ± 12.1	130.8 ± 34.8/22.3 ± 14.8	*0.039*
PWV (m/sec) (mean ± SD)	14.3 ± 3.8	11.9 ± 2.6	9.2 ± 1.9	<*0.0001*
RHI (mean ± SD)	1.6 ± 0.4	1.8 ± 0.5	2.4 ± 0.6	<*0.0001*
MMSE (mean/sd)	26.5 ± 3.6	27.42 ± 2.3	29.2 ± 1.1	<*0.0001*
(HbA1c) (%) (mean/sd)	8.2 ± 1.3	7.3 ± 1.1	–	<*0.0001*
Antihypertensive drugs (%/n)				
Ace-inhibitors	(26/52)	23 (46)	0	<*0.0001*
*ARBs*	13 (26)	10 (20)	0	<*0.0001*
(CCB)	10 (20)	9 (18)	0	<*0.0001*
Statins (%/n)	29 (58)	28 (56)	0	<*0.0001*
Antidiabetic drugs (%/n)				
Sulfonylureas	23 (46)	21 (42)	0	<*0.0001*
*Metformin*	22 (44)	23 (46)	0	<*0.0001*
Insulin	38 (76)	31 (62)	0	<*0.0001*

*BMI* body mass index, *BSA* body surface area, *SBP* systolic blood pressure, *DBP* diastolic blood pressure, *Aortic AIx* aortic augmentation index, *AP* augmentation pressure, *PWV* pulse wave velocity, *RHI* reactive hyperaemia index, *MMSE* mini mental state examination, *HbA1c* glycated haemoglobin, *ARBs* angiotensin receptor 1 blockers, *eGFR* estimated glomerular filtration rate according Cockcroft and Gault formula, *CCB* calcium channel blockers

Italic values indicate significance of p value (p < 0.05)

In comparison to diabetic subjects without diabetic foot and healthy subjects, the subjects with diabetic foot also had higher mean values of PWV (14.3 ± 3.8 vs. 11.9 ± 2.6 vs. 9.2 ± 1.9 m/sec; P < 0.0001), lower mean value of RHI (1.6 ± 0.4 vs. 1.8 ± 0.5 vs. 2.4 ± 0.6; P < 0.0001), lower mean value of MMSE (26.5 ± 3.6 vs. 27.4 ± 2.26 vs. 29.2 ± 1.1; P < 0.0001). No significant difference was found between subjects with diabetic foot and diabetic subjects without diabetic foot (6.1 ± 1.3 years vs. 6.5 ± 2.2 years; P = 0.56).

At multinomial logistic regression analysis of variables independently associated with diabetes and diabetic foot presence, we found that age (OR: 1.06; P = 0.030), BMI (OR: 1.21; P = 0.002), PWV (OR: 1.60; P = 0.014), RHI (OR: 0.31; P = 0.043) and MMSE (OR: 0.17; P = 0.036) were associated with diabetes, whereas hypertension (OR: 21.3; P < 0.0001), Dyslipidaemia (OR: 6.1; P < 0.014), BMI (OR: 1.2; P = 0.019), PWV (OR: 2.26; P = 0.002), RHI (OR: 0.011; p = 0.002) (see Table 2) were associated with diabetic foot presence.

**Table 2:** Multinomial logistic regression analysis of variables predictive of diabetes and diabetic foot presence Odds ratios (OR) were adjusted for drug therapy as covariate

Variables	OR	95% Confidence interval	**p**
Diabetes			
Hypertension	3.68	0.96–14.03	0.056
Dyslipidaemia	2.80	0.76–10.27	0.120
Age	1.06	1.02–1.12	*0.030*
BMI	1.21	1.07–1.36	*0.002*
SBP	0.97	0.93–1.01	0.253
Aix	1.0	0.96–1.04	0.887
PWV	1.60	1.09–2.33	*0.014*
RHI	0.31	0.10–0.96	*0.043*
MMSE	0.17	0.35–0.89	*0.036*
Diabetic foot			
Hypertension	21.27	4.09–110.62	*0.0001*
Dyslipidaemia	6.07	1.43–25.66	*0.014*
Age	1.0	0.94–1.07	0.901
BMI	1.17	1.02–1.34	*0.019*
SBP	0.99	0.95–1.04	0.936
AIx	1.01	0.90–1.14	0.757
PWV	2.26	1.36–3.75	*0.002*
RHI	0.01	0.001–0.185	*0.002*
MMSE	0.27	0.04–1.79	0.176

*BMI* body mass index, *SBP* systolic blood pressure, *DBP* diastolic blood pressure, *Aortic AIx* aortic augmentation index, *AP* augmentation pressure, *PWV* pulse wave velocity, *RHI* reactive hyperaemia index, *MMSE* mini mental state examination

Italic values indicate significance of p value (p < 0.05)

**Fig. 1:**
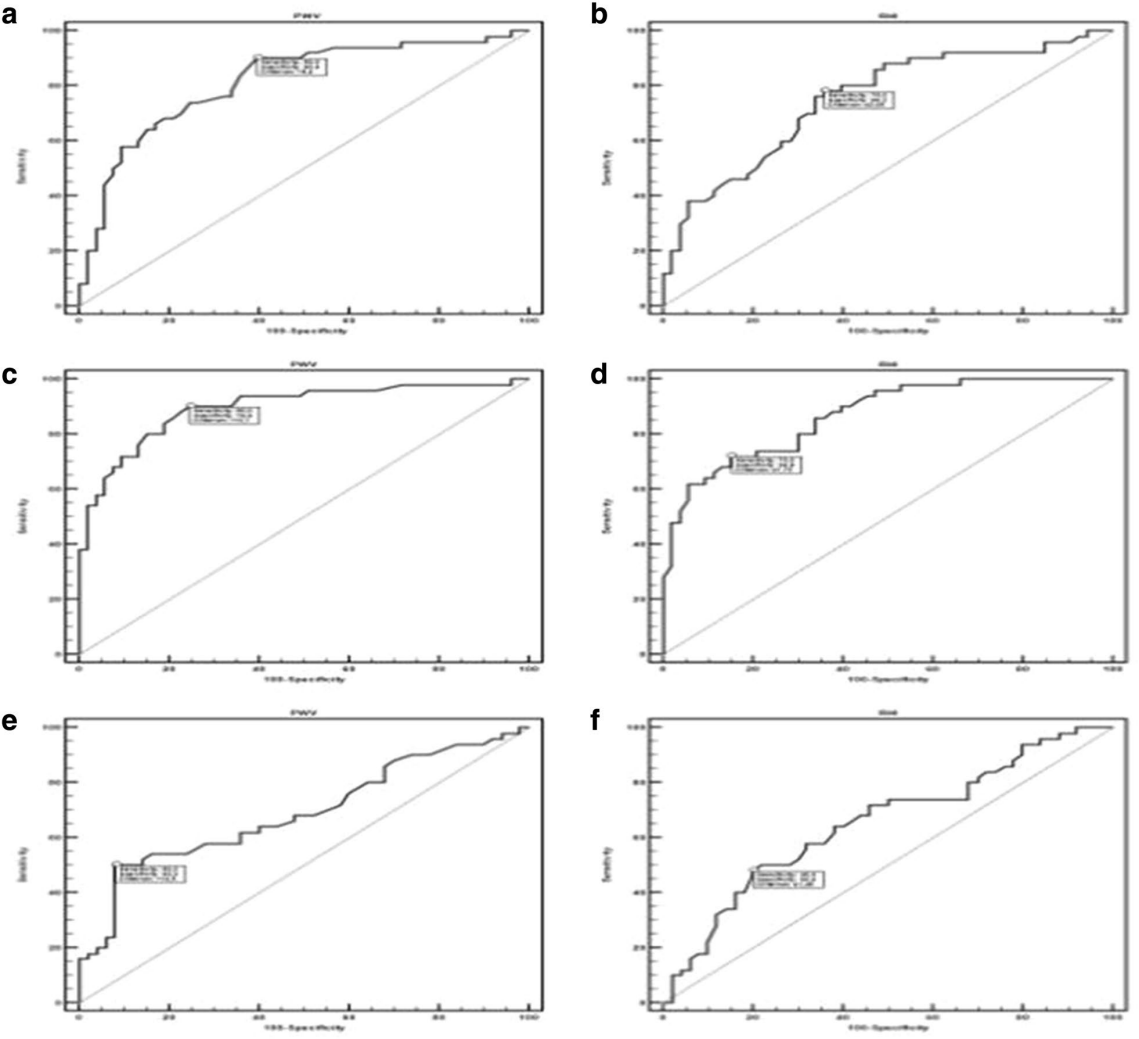
Area under ROC *curve*, sensitivity and specificity of PWV, Aix and RHI towards diabetes and diabetic foot (**a**–**f**). **a** Area under ROC curve, sensitivity and specificity of pulse *wave* velocity (PWV) in diabetic subjects vs. controls. **b** Area under ROC curve, sensitivity and specificity of ractive hyperaemia index (RHI) in diabetic subjects vs. controls **c** Area under ROC *curve*, sensitivity and specificity of pulse *wave* velocity (PWV) in subjects with diabetic foot vs. controls. **d** Area under ROC curve, sensitivity and specificity of ractive hyperaemia index (RHI) in subjects with diabetic foot vs. controls. **e** Area under ROC *curve*, sensitivity and specificity of pulse *wave* velocity (PWV) in subjects with diabetic foot vs. diabetics. **f** Area under ROC *curve*, sensitivity and specificity of ractive hyperaemia index (RHI in subjects with diabetic foot vs. diabetics

Moreover by means of ROC curve analysis there was good sensitivity and specificity of arterial PWV (AUC = 0.816, P < 0.0001; cutoff value ≥9.4 m/sec, sensitivity = 90, specificity = 60.4) and RHI (AUC = 0.751, P < 0.0001; cutoff value ≤ 2.05, sensitivity = 78, specificity = 64.2) for diabetes presence compared to healthy subjects (see Fig. 1).

On ROC curve analysis there was also good sensitivity and specificity of arterial PWV (AUC = 0.897, P < 0.0001; cutoff value ≥ 10.1, sensitivity = 90, specificity = 75.5) and RHI (AUC = 0.869, P < 0.0001; cutoff value ≤1.72, sensitivity = 72, specificity = 84.9) for diabetic foot presence compared to healthy subjects, and PWV (AUC = 0.690, P = 0.003; cutoff value ≥ 14.5 m/sec, sensitivity = 50, specificity = 92) and RHI (AUC = 0.654, P = 0.0049; cutoff value ≤ 1.45 m/sec, sensitivity = 48, specificity = 80) predicted diabetic foot compared to diabetic subjects (see Fig. 1).

**Fig. 2:**
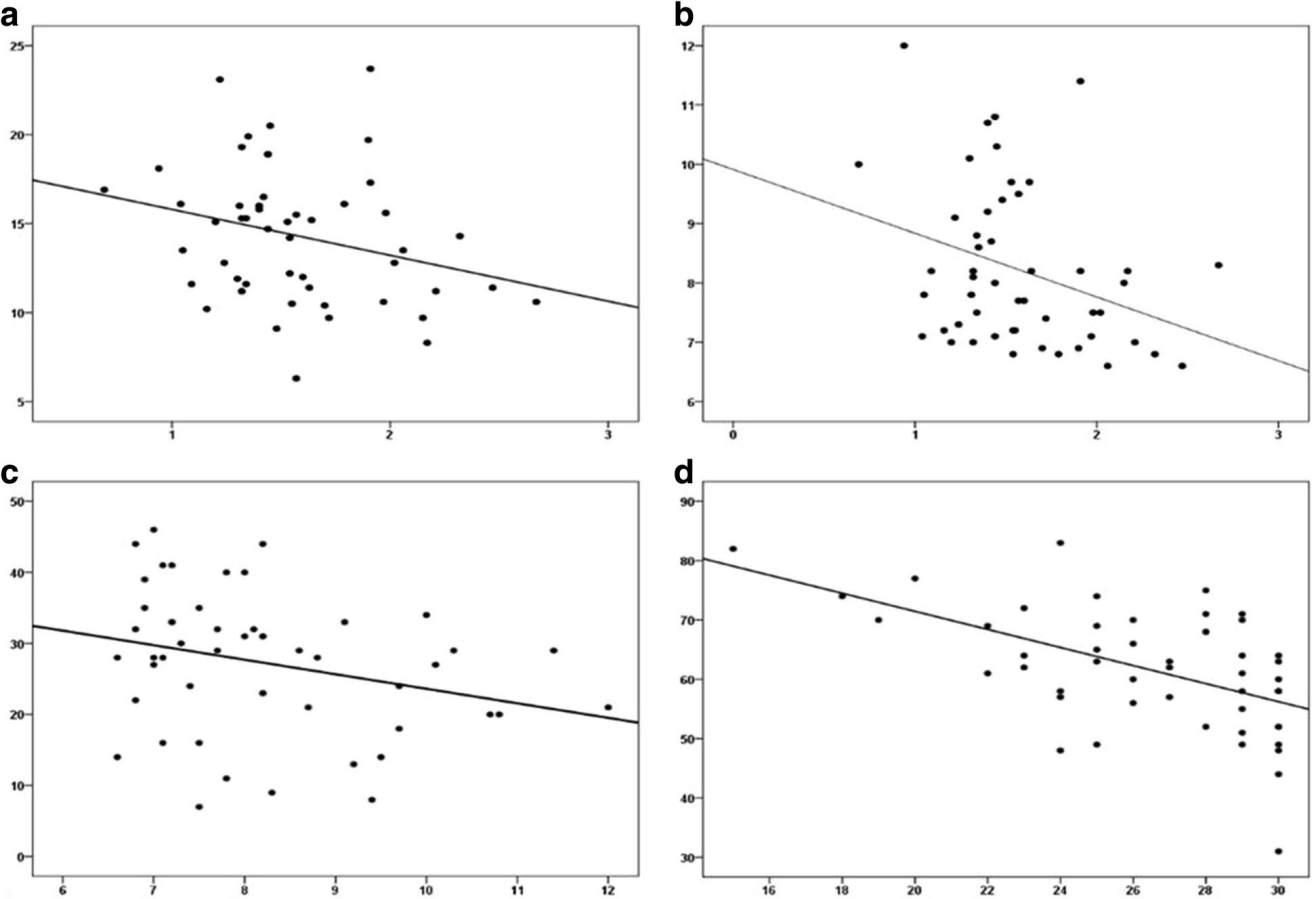
Significant correlations among clinical and arterial stiffness and endothelial function variables (**a**–**c**). **a** Correlation between PWV and RHI (reactive hyperaemia index); **b** correlation between RHI and hemoglobin A1c (HbA1c); **c** correlation between augmentation index (AIx) and hemoglobin A1c; **d** correlation between minimental state examination (MMSE) score and age

In subjects with diabetic foot on correlation analysis we observed a significant negative correlation between PWV and RHI (r = −0.31, P = 0.029), a significant negative correlation between RHI and previous cardiovascular events (r = −0.30, P = 0.029) and glycated hemoglobin (HbA1c) (r = −0.30; P = 0.030), and between MMSE and age (r = −0.50, P < 0.0001), dyslipidaemia (r = −0.31; P = 0.028) and previous cardiovascular events (r = −0.37; P = 0.007) (see Fig. 2).

We subdivided patients with diabetes and diabetic foot regarding HbA1c (first tertile: HbA1c <7%, second tertile HbA1c >7%; third tertile HbA1c >10%. No significant correlation was observed among patients with diabetes without diabetic foot and diabetic patients with diabetic foot between HbA1C tertiles and PWV (P = 0.104), RHI (P = 0.463), Aix (P = 0.294), whereas a significant negative correlation was observed between MMSE and HbA1C tertiles (R = 0.316; p = 0.037).

## Discussion

In comparison to control diabetic subjects without diabetic foot and healthy controls we found that subjects with DFS had higher mean values of PWV, lower mean values of RHI and lower mean MMSE. To the best of our knowledge, only a few studies to date have evaluated vascular health markers such as arterial stiffness and endothelial function indexes in diabetic foot subjects compared to diabetic subjects without foot complications and in healthy controls. Katakami et al. [25] recently showed that evaluation of baPWV, in addition to carotid IMT and conventional risk factors, improved the ability to identify the diabetic individuals with high risk for CVE, whereas very recently Gomez-Marcos et al. [26] analyzed the relationship between cardio ankle vascular index (CAVI), a new index of the overall stiffness, and target organ damage (TOD), vascular structure and function, and cardiovascular risk factors in Caucasian patients with type 2 diabetes mellitus or metabolic syndrome, suggesting that CAVI is positively associated with IMT, cf-PWV, ba-PWV, CAIx, and PAIx, regardless of cardiovascular risk and the drug treatment used.

Siasos et al. [27] reported a significant association between diabetic microvascular complications such as diabetic retinopathy and vascular dysfunction, indicating that these conditions coexist in diabetic patients. In another study Antonopoulos et al. reported [28] that both brachial FMD and AIx are strongly and independently associated with DFS.

Nevertheless, to our knowledge no study has yet examined endothelial function by means of reactive hyperemia peripheral arterial tonometry (RH-PAT), thus our finding appears original. The main consequence of endothelial dysfunction is the initiation of an inflammatory process which leads to the formation of atherosclerosis and its late sequel, cardiovascular morbidity and mortality. Because of its central role in mediating vessel tone and growth, its position as gateway to circulating immune cells, and its local regulation of hemostasis and coagulation, the properly functioning endothelium is the key to cardiovascular health. Diabetic foot syndrome is a micro and macrovascular complication of diabetes, and loss of this vasodilator mechanism may contribute to cardiovascular morbidity such as disordered coronary flow regulation [29]. Furthermore, early stages of epicardial atherosclerosis are associated with an impairment in endothelium-dependent dilation of the coronary microvasculature, indicating that the pathophysiological consequences of atherosclerosis may extend into the human coronary microcirculation [30].

Thus both microvascular and macrovascular complications of diabetes may be fully depicted by means of evaluation of endothelium-dependent vasodilation indexes such as the PAT index RHI.

A previous study conducted by our group [12] reported that in comparison to diabetics without diabetic foot, subjects with diabetic foot had higher IL-6 and resitin plasma levels, and lower adiponectin plasma levels. These previous findings could represent a possible pathogenic explanation of higher arterial stiffness indexes and lower mean values of an endothelial function index such as the RHI reported by our current study.

Resistin contributes to insulin resistance and it may also regulate inflammatory responses [31]. Moreover, Osawa et al. [32] reported that elevated serum resistin concentration appears to be an independent risk factor for ischemic stroke, especially lacunar and atherothrombotic infarction in the Japanese general population. The candidate role of hypo-adiponectinemia as a putative marker of cardiovascular morbidity may explain our findings of a higher degree of arterial stiffness and a low degree of endothelial function indexes in subjects with diabetic foot. Since adipose tissue produces several cytokines [33], an “adipo-vascular” axis [34] may represent the pathogenic basis of the increased risk of cardiovascular events in patients with type 2 diabetes, which is also well expressed by the involvement of surrogate cardiovascular markers such as arterial stiffness and endothelial function indexes. In patients with diabetic foot lower plasma levels of adiponectin and higher plasma levels of IL-6 and resistin could be linked to endothelial dysfunction and progressive arterial stiffening. Recent studies suggest that adiponectin may play a role in the modulation of inflammatory vascular response by inhibiting the expression of adhesion molecules on endothelial cells [35], inhibiting endothelial cell NF-kB signaling [36] and suppressing macrophage function [37]. Other studies showed that adiponectin suppressed the TNF-a-stimulated expression of E-selectin, VCAM-1 and ICAM-1 in human endothelial cells [38]. Adiponectin elicits mainly endothelium-dependent dilation of the retinal arterioles [39]. Endothelium-dependent vasodilation likely induced by adiponectin results from NO via activation of guanylyl cyclase that is partially dependent on AMPK activity, thus low serum levels of adiponectin may explain our findings of a lower RHI index in subjects with diabetic foot. Our findings concerning significantly lower mean values of RHI and higher mean values of arterial stiffness indexes may also contribute to explaining our findings of lower cognitive performance in subjects with diabetic foot.

Few studies have addressed the interplay between arterial stiffness, type 2 diabetes, and cognitive performance. A recent study [40] reported that carotid stiffness is associated with cognitive performance in both individuals with and without diabetes, but does not mediate the relationship between type 2 diabetes and cognitive dysfunction. The role of primitive vascular alterations in the development of cognitive decline is a major issue in both research and clinical practice. In a recently published cross-sectional study [29] conducted on the “ADELAHYDE” cohort comprised of older hypertensive patients with memory complaints showing the association of arterial changes (hypertrophy and arterial stiffness, endothelial dysfunction) with cognitive functions and/or white matter hyper intensities on MRI, the role of vascular factors in the evolution of cognitive function and onset of dementia has been confirmed.

Thus vascular abnormalities, independently of blood pressure levels, may play a role in the setting of subjective memory complaints as well as white matter hyper intensities (WMH) in elderly hypertensive patients. Arterial thickness and stiffness as well as endothelial function should be assessed simultaneously and may represent additional targets for the prevention of subjective memory complaints and WMH. We have not reported mean MMSE values indicating clear cognitive dysfunction in both groups of diabetic subjects but our findings of lower MMSE mean scores observed in diabetic subjects with diabetic foot may represent a further issue confirming the role of arterial stiffness and endothelial dysfunction in the clinical setting of cognitive performance as well. Nevertheless our finding of an MMSE value of 26.5 ± 3.6 in subjects with diabetic foot may suggest more impaired cognitive performance in subjects with a higher degree of arterial stiffness and a lower degree of endothelial mediated vasodilation. Consistent with our findings a recent study [41] conducted to evaluate the utility of MMSE scores in detecting cognitive dysfunction in a sample of highly educated individuals showed that an MMSE cutoff score of 27 resulted in an optimal balance of sensitivity and specificity with an overall correct classification rate of 90% in a cognitively impaired group (dementia and MCI), thus indicating that a cutoff score of 27 might be more appropriate.

We previously reported [11] that diabetic patients with diabetic foot were more likely to have a higher prevalence of cardiovascular risk factors, a higher prevalence of previous cardiovascular morbidity (coronary artery disease, transient ischemic attack/ischemic stroke, diabetic retinopathy), and a higher prevalence of subclinical cardiovascular disease. Furthermore, diabetic patients with foot ulceration had a higher incidence of new-onset vascular events (coronary artery disease, transient ischemic attack/ischemic stroke, diabetic retinopathy) on a 5-year follow-up.

Thus our findings of a significantly lower mean MMSE, consistent with our other findings concerning arterial stiffness and endothelial indexes in diabetic subjects with DFS could be related to the higher degree of cardiovascular and cerebrovascular risk and more advanced cerebral vascular disease both involving brain microvessels and brain white matter. The pathogenesis of cerebral small vessel disease, which involves white matter lesions (WMLs) and cerebral microbleeds (CMBs), is thought to be associated with endothelial dysfunction [42, 43].

In a mouse model of mice with genetically greater large artery stiffness a recent study [44] that examined the cause-and-effect relationship between large artery stiffness and peripheral resistance artery function indicated the presence of an impaired cerebral artery endothelial function. Another recent study [45] aimed to determine the associations between central hemodynamics and brain structure at rest and during exercise in people with and without T2DM, reporting that brain atrophy is associated with resting aortic stiffness in T2DM, and that central vascular mechanisms underlying structural brain changes may differ between healthy individuals and T2DM. These findings could also represent a further pathogenic issue of cognitive impairment in human subjects with a higher degree of arterial stiffness.

Cognitive deficits in T2DM may also be partly attributable to stiffness in cerebral arteries and impaired vasodilator function, limiting the ability to increase blood flow in brain regions to meet cognitive demands. Nealon et al. [46] recently compared cerebrovascular responsiveness (CVR) and cognitive performance in adults with and without T2DM by means of measurements of basal cerebral mean blood flow velocity (MBFV) and pulsatility index (PI), a measure of arterial stiffness in the middle cerebral arteries (MCA) showing that PI was higher in the T2DM group with lower cognitive performance, and that cognitive function was inversely related to the PI/MBFV ratio, an indicator of intracranial stenosis.

Our findings on multinomial analysis concerning the predictive role of PWV, RHI and MMSE for diabetes presence, and PWV and RHI for diabetic foot presence are consistent with the higher degree of vascular impairment of diabetic subjects and in particular diabetic subjects with DFS. Arterial stiffness and endothelial function indexes are important surrogate markers that describe the capability of an artery to expand and contract in response to pressure changes and other stimuli. Thus a worse endothelial, arterial stiffness and cognitive profile of patients with diabetic foot could be a good marker of a higher degree of vascular involvement consistent with higher cardiovascular morbidity and metabolic cardiovascular pathogenic pathways of higher IL-6 and resistin plasma levels, and lower adiponectin plasma levels as previously reported [12] in subjects with diabetic foot. Correlation analysis findings between arterial stiffness, endothelial function and cognitive markers and other clinical and laboratory variables help sketch the cardiovascular risk profile of subjects with diabetic foot. In subjects with diabetic foot we observed a significant negative correlation between RHI values and previous cardiovascular events, HBA1C and PWV, and a significant negative correlation between MMSE and age, dyslipidaemia and previous cardiovascular events. These findings highlight the role of the cardiovascular and metabolic background of patients with diabetic foot in the pathogenesis of vascular and cognitive surrogate markers of vascular impairment, also suggesting a common mechanism of pathways for microvascular and macrovascular disease in diabetic subjects with foot complications.

Diabetes mellitus leads to accelerated progression of arteriosclerosis with an increased risk of coronary events in comparison to non-diabetic patients. A recent study [47] measured flow-mediated dilation (FMD) of the brachial artery non-invasively as a marker for endothelial function, fractional diameter changes (FDC) as a marker for physical–mechanical properties, intima-media thickness (IMT) as a marker for structural properties, and forearm blood flow (FBF) as a marker for microvascular function. This study reported that diabetes was associated with reduced FMD indicating impaired macrovascular endothelial function and parallel reduced FDC and increased IMT, indicating increased stiffness and enhanced structural alterations and reduced forearm blood flow during reactive hyperemia indicating microvascular dysfunction.

One common pathogenic mechanism for microvascular disease is rooted in chemical reactions between by-products of sugars and proteins that occur over the course of days to weeks and eventually produce irreversible cross-linked protein derivatives called AGE [48] These derivatives can exhibit a wide range of effects on surrounding tissues, including modification (e.g. thickening) of collagen [49] and endothelium [50–53]. Nevertheless the pathogenesis of macrovascular disease in diabetes is multifactorial; the common recipient of injury is the vascular endothelium since diabetes initially impairs the ability of the vascular endothelium to vasodilate through inhibition of the nitric oxide [54]. The vascular endothelium also loses its ability to produce NO-activated tissue plasminogen activator, a fibrinolytic (anti-clotting) protein that inhibits the ability of inflammatory cells to “stick” to the endothelial surface [55, 56]. Insulin resistance can also contribute to a decrease in NO production and the subsequent impaired vasodilatory response. In addition to the reduction in the vasodilatory response in diabetes, an overproduction of vasoconstriction substances occurs; these substances include endothelin 1, which has direct vasoconstrictive effects on the endothelium as well as indirect fluid volume effects, including the stimulation of water and salt retention and the activation of the RAS. Both of these pathogenic pathways seem to be suggested by many of our findings, such as impaired arterial stiffness indexes and RHI indexes, and these vessel changes may also explain the worse cognitive profile of subjects with diabetic foot in comparison to diabetic patients without foot complications and healthy subjects.

### Limitations

We have not evaluated the prevalence of WMLs in our enrolled subjects thus avoiding an analysis of possible involvement of brain white matter disease in determining the lower cognitive performance of patients with diabetic foot. The cross-sectional analysis is a limitation since it does not allow conclusions on cause-effect relationships and the relatively small sample size may also limit conclusions. Nevertheless a future study conducted by our group will evaluate the prevalence of WMLs in subjects with diabetic foot in comparison to control subjects without diabetic foot.

## Conclusions

This study reports that in comparison to controls, subjects with diabetic foot had (1) higher mean levels of pulse wave velocity and augmentation index, and significantly lower mean RHI values; (2) on multinomial logistic regression analysis PWV and RHI were significantly predictive of diabetic foot diagnosis; (3) ROC curve analysis had good sensitivity and specificity of arterial stiffness and PAT indexes to predict diabetic foot; (4) on correlation analysis a significant negative correlation between PWV and RHI, RHI and previous cardiovascular events (HB1ac and between MMSE and age, dyslipidaemia, previous cardiovascular events). Future research could address these issues by means of longitudinal studies to evaluate cardiovascular event incidence in relation to arterial stiffness, endothelial and cognitive markers, and to address the beneficial effects of cardiovascular drugs such as statins and ACE inhibitors on these surrogate markers.

## Abbreviations

MMSE: Mini Mental State Examination
PWV: pulse wave velocity
Aix: augmentation index
RH-PAT: reactive hyperemia-peripheral arterial tonometry
RHI: reactive hyperemia index
DFD: Diabetic foot disease
Alb: albuminuria
DR: diabetic retinopathy
CVD: cardiovascular diaease
ESC-ESH: European Society of Cardiology-European Society of Hypertension
TG: triglycerides
HDL: high density lipoprotein
BMI: body mass index
ALT: alanine transaminase
PP: pulsatory pressure
PAT: peripheral arterial tonometry
ANOVA: univariate analysis of variance
CIs: confidence intervals
ROC: receiver operating characteristic AUC: area under the curve
FMD: flow-mediated dilation
T2DM: type 2 diabetes mellitus
TIA: transient ischemic attack
WMH: white matter hyperintensities
HBA1C: glycated hemoglobin
AGE: Advanced glycation end products
NO: nitric oxide
WMLs: White matter lesions
